# A Comprehensive Review of Performance of Polyacrylonitrile-Based Membranes for Forward Osmosis Water Separation and Purification Process

**DOI:** 10.3390/membranes13110872

**Published:** 2023-11-03

**Authors:** Nada Abounahia, Arqam Azad Shahab, Maryam Mohammad Khan, Hazim Qiblawey, Syed Javaid Zaidi

**Affiliations:** 1 UNESCO Chair in Desalination and Water Treatment, Center for Advanced Materials (CAM), Qatar University, Doha P.O. Box 2713, Qatar; 2 Department of Chemical Engineering, College of Engineering, Qatar University, Doha P.O. Box 2713, Qatar; hazim@qu.edu.qa

**Keywords:** polyacrylonitrile, forward osmosis, membranes, phase inversion, electrospinning, nanofiber, water, separation

## Abstract

Polyacrylonitrile (PAN), with its unique chemical, electrical, mechanical, and thermal properties, has become a crucial acrylic polymer for the industry. This polymer has been widely used to fabricate ultrafiltration, nanofiltration, and reverse osmosis membranes for water treatment applications. However, it recently started to be used to fabricate thin-film composite (TFC) and fiber-based forward osmosis (FO) membranes at a lab scale. Phase inversion and electrospinning methods were the most utilized techniques to fabricate PAN-based FO membranes. The PAN substrate layer could function as a good support layer to create TFC and fiber membranes with excellent performance under FO process conditions by selecting the proper modification techniques. The various modification techniques used to enhance PAN-based FO performance include interfacial polymerization, layer-by-layer assembly, simple coating, and incorporating nanofillers. Thus, the fabrication and modification techniques of PAN-based porous FO membranes have been highlighted in this work. Also, the performance of these FO membranes was investigated. Finally, perspectives and potential directions for further study on PAN-based FO membranes are presented in light of the developments in this area. This review is expected to aid the scientific community in creating novel effective porous FO polymeric membranes based on PAN polymer for various water and wastewater treatment applications.

## 1. Introduction

The forward osmosis (FO) process has received considerable research attention in recent years and has been successfully applied in numerous applications, including desalination of seawater and brackish water, wastewater treatment and purification, the concentration and separation of food and pharmaceuticals, as well as other new fields [1,2]. This high interest is due to its simplicity, low cost, low energy consumption, and minimal tendency of membrane fouling compared to other pressure-driven membrane-based technologies such as reverse osmosis (RO) and nanofiltration (NF) [3,4]. FO process is a concentration-driven process in which water is transferred from the feed solution to the draw solution across a semi-permeable membrane, without requiring high hydraulic pressure [3].

The membrane is a crucial component of the FO process. The membrane modules, and membrane properties play a major role in FO performance and applicability. Selecting proper production and modification procedures can result in high-performance membranes. The optimum membrane for the FO water treatment process must have high water permeability and solute retention, as well as excellent chemical stability, high mechanical strength, low concentration polarization, and low fouling tendency. Thus, achieving all these desired properties has become an important research focus of FO membrane development by various research groups [5,6,7,8]. Thin-film composite (TFC) membranes have been created for FO to further increase membrane performance. TFC membranes have an asymmetric structure with one top selective skin and a porous support layer. Most of the TFC-FO membranes were fabricated on a lab scale with different materials using the phase inversion method, interfacial polymerization (IP) technique, electrospinning, and layer-by-layer (LBL) deposition [5]. 

Over the past few years, numerous research studies by both academia and industry have been conducted on FO membrane fabrication and modification, using various polymers to achieve the best FO separation and selectivity performance. The most extensively utilized polymers for the preparation of the FO membrane support layer include cellulose acetate/triacetate (CA/CTA) [9], polyvinylidene fluoride (PVDF) [10], polyacrylonitrile (PAN) [11], polyethersulfone (PES) [12], polysulfone (PSf) [13], and so on. In a number of review studies, it was found that the selection of the polymer solution and its rheological properties throughout membrane preparation play a crucial role in defining membrane morphology and performance [14]. 

Among various polymers, PAN polymer, with its unique characteristics such as low material cost, outstanding physicochemical properties, and wide commercial availability, has sparked a lot of research interest in FO membrane fabrication at the laboratory scale [15]. Most of the PAN-based porous FO membranes were fabricated at a lab scale mainly via the phase inversion technique [16], and a few membranes were prepared via the electrostatic spinning method [17]. Moreover, these membranes were tested under FO conditions after being modified using nanomaterials as membrane fillers to improve their performance in terms of flux, rejection, and resistance to fouling [18,19,20,21,22,23].

To the best of our knowledge, no comprehensive review of the use of PAN-based membranes in FO operation tests has been conducted. Therefore, this work aims to review all of the PAN-based porous FO membranes that were previously used, considering their fabrication, modification techniques, and FO performance results. Our research is anticipated to be beneficial to this field’s researchers by providing new avenues for the successful development of new porous FO polymeric membranes based on PAN polymer.

## 2. Forward Osmosis Process and Polymeric Membranes

Osmosis is a natural process that experts in many fields of science and engineering have examined in depth. The osmosis phenomenon applied to natural materials was first examined by researchers, and starting in the 19th century, osmosis received particular attention [24]. Osmosis, or forward osmosis as it is currently known, offers a wide range of possible uses, including wastewater treatment, power production, desalination, and food processing. When associated with other membrane processes that utilize pressure, FO is receiving more and more attention. As opposed to pressure-driven membrane processes, FO provides numerous advantages, including less energy consumption, minimal fouling propensity, simple or low maintenance, and reduced costs. In addition, FO is receiving more attention for wastewater treatment than other approaches, including solvent extraction, ion exchange, absorption, biosorption, and so on [25,26,27,28,29], because the high cost of chemicals and equipment prevents solvent extraction from being used on a large scale [29]. In addition, despite the fact that ion exchange and adsorption have garnered much attention for their excellent selectivity, simple operation, and strong thermal and radiation stabilities [28], choosing a promising resin or adsorbent with high capacity and selectivity, a high adsorption rate, and low cost remains a difficulty [27].

In FO, two solutions, a concentrated draw solution and a more diluted feed solution, are positioned within a semipermeable membrane [30]. FO, in general, is a mixing and separating process. The draw solution’s chemical potential is decreased by the water molecules that combine with the feed solution when they cross the membrane from a diluted feed solution, motivated by the natural osmotic pressure difference between the two solutions. Water permeates the membrane until equilibrium is established, and the osmotic pressures of the FS and DS solutions are equal [31,32].

Several drawbacks of other membrane technologies using pressure, for example, RO, can be addressed by FO through utilizing the osmotic pressure differential to stimulate water through the membrane [33]. There are several factors that could affect the performance of the FO desalination process, such as feed and draw solution properties and operating parameters, including crossflow rates and temperature [34]. The water flux was observed to increase slightly at higher crossflow rates. However, the effectiveness of the FO process was shown to be significantly influenced by temperature. The most obvious effect of increasing the system temperature is an increase in water flux over the membrane because of decreased water viscosity and increased water diffusivity, which together effectively increase water permeability across the membrane [35].

**Figure 1:**
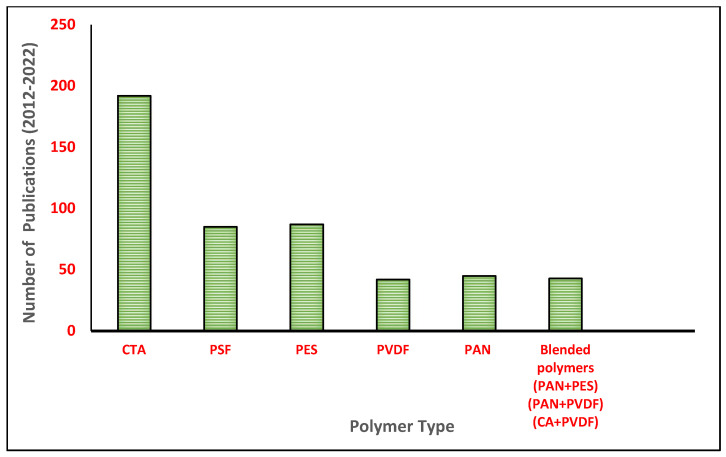
The number of studies published in the FO literature using different polymeric membranes. Scopus was used to retrieve the data by searching for articles with a polymer name and “Forward osmosis” in the title’s keywords.

**Figure 2:**
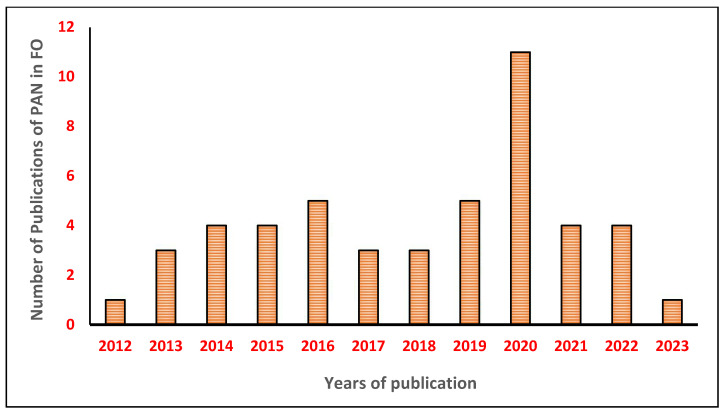
The number of studies published in the FO literature using PAN membranes. Scopus was used to retrieve the data by searching for articles with “Polyacrylonitrile” and “Forward osmosis” in the title’s keywords.

On the other hand, the membrane represents a crucial part of the FO process, and its properties could affect process efficiency [2]. It can be fabricated using many types of polymers, including cellulose triacetate [36,37,38,39,40], polyethersulfone [41,42,43], polysulfone [44,45,46], polyvinylidene fluoride [47,48,49,50], polyacrylonitrile [51,52], and so on. As can be seen in Figure 1 below, cellulose triacetate is the most commonly utilized form of polymer material, and polyvinylidene fluoride is the least common. CTA is widely used because of its strong chlorine resistance, propensity to resist fouling, low cost, and easy accessibility [36,37,38,39,40]. The FO membrane in general must be hydrophilic, have strong mechanical strength, and have outstanding thermal and chemical resistance in order to achieve optimum FO performance. In some studies, blended polymers have also been utilized to enhance the forward osmosis membrane properties [53,54,55]. Polymers are blended because combining two or more polymers will create a membrane substrate that might, in theory, have a mutually beneficial property where the strength of one material can compensate for the weakness of another. Among blended polymers for FO, the superior thermal, mechanical, and chemical qualities of PVDF can be considered. However, PVDF’s primary flaw is that it is a hydrophobic polymer. To circumvent this problem, PVDF can be combined or blended with the hydrophilic, stretchable, spinnable, and inexpensive PAN polymer which will provide an overall benefit to the FO process [55]. However, as shown in Figure 2, there is a rising interest in PAN after 2018, between 2019–2021. This is mainly due to the PAN polymer having superior characteristics, which make it desirable for FO operation. Recent researchers in 2020, have realized the capability of PAN membranes to elevate FO performance, as PAN has several benefits, including hydrophilicity [53], comparatively good chemical and thermal stability, particularly resistance to active chlorine, stability in a medium of nonpolar and low-polar organic solvents, such as hydrocarbons, alcohols, and esters, and is considerably cheap [56]. Moreover, PAN is also attractive as the support layer in the membranes due to its superior chemical and solvent stability, and high hydrophilicity; hence, it is possible to use TFC membranes in a wider range of advanced membrane processes, including forward osmosis and organic solvent nanofiltration. Additionally, PAN’s excellent chemical tunability (such as surface hydrophilicity and charge density) via hydrolysis allows for the strong creation of the polyelectrolyte interlayer on top of it by managing electrostatic contact [57].

Depending on the applications for the forward osmosis process, fabrication methods and membrane materials can be altered and improved. Desalination and wastewater treatment are the two principal uses of forward osmosis [52]. Different FO-based membranes can be made in many ways, including commercially, through phase inversion, electrospinning, and interfacial polymerization [38,40,44,45].

## 3. PAN Chemical Structure and Characteristics as Membrane Substrate

**Figure 3:**
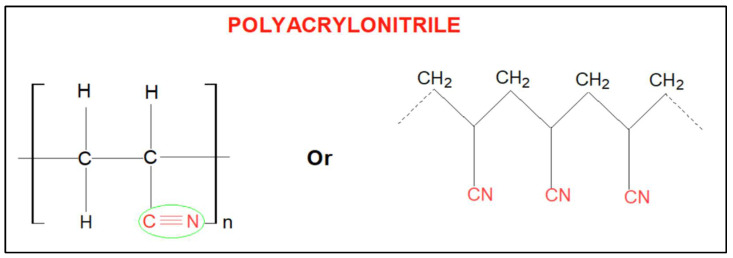
The general molecular structure of polyacrylonitrile.

Polyacrylonitrile has been widely used as a substrate for various membranes like UF, microfiltration (MF), NF, and RO because of its good physiochemical properties such as high chemical stability, solubility to common solvents (e.g., N-methyl-2-pyrrolidone, dimethylformamide (DMF), dioxanone, chloroacetonitrile, dimethyl phosphite, etc.), and high hydrophilicity in comparison to other membrane materials such as polysulfone, polyethersulfone, polyethylene, and polypropylene [58,59]. This high hydrophilicity refers to its higher molecular polarity, attributed to the existence of polar nitrile groups (–C≡N) in PAN molecules, as shown in Figure 3 [60]. In addition, the triple bonds of the nitrile groups have the potential to be further converted into more hydrophilic segments through, for example, the alkaline hydrolysis reaction [15]. PAN polymer has a decomposition temperature >175 °C [59]. Moreover, compared to other polymer materials, PAN has good resistance against chlorine and multiple chemicals. As a result, it has been known as a low-fouling membrane for aqueous filtration and has already been commercialized [61,62]. In addition, it is worth mentioning that the transport properties of PAN-based membranes could also be adjusted by PAN copolymers containing the new N-methylmorpholine-N-oxide (NMMO) solvent, which enables the quick creation of highly concentrated PAN solutions [63,64,65].

## 4. PAN Pretreatment Surface Modification Techniques

**Figure 4:**
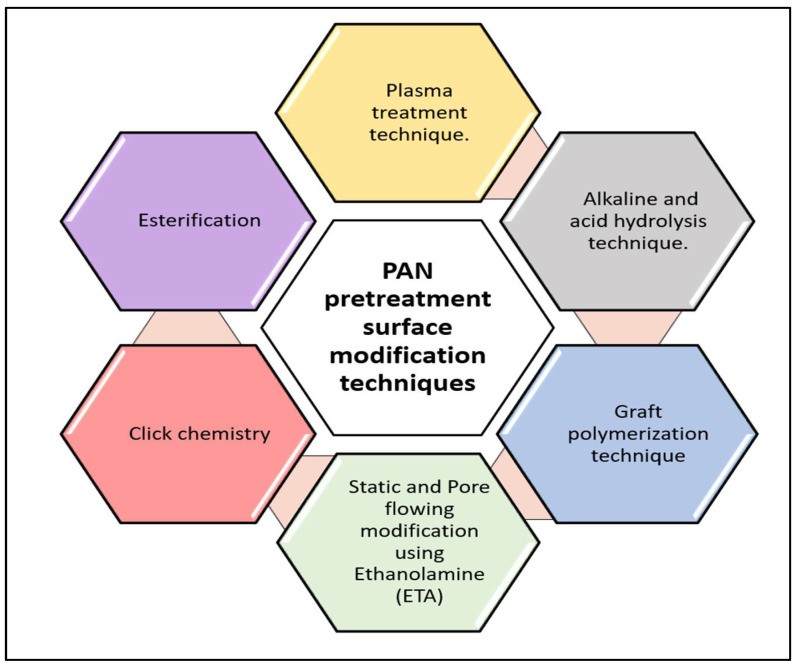
PAN pretreatment surface modification techniques.

PAN is a relatively active polymer, which makes it easy to alter and modify. As shown in Figure 4, there are various surface modification techniques for PAN-based membranes. Plasma treatment and chemical treatment (hydrolysis technique) are the most common PAN membrane modification procedures to alter its antifouling properties and surface properties, such as hydrophobicity, chemical structures, and roughness [11,66,67,68,69].

### 4.1. Plasma Treatment Technique

**Figure 5:**
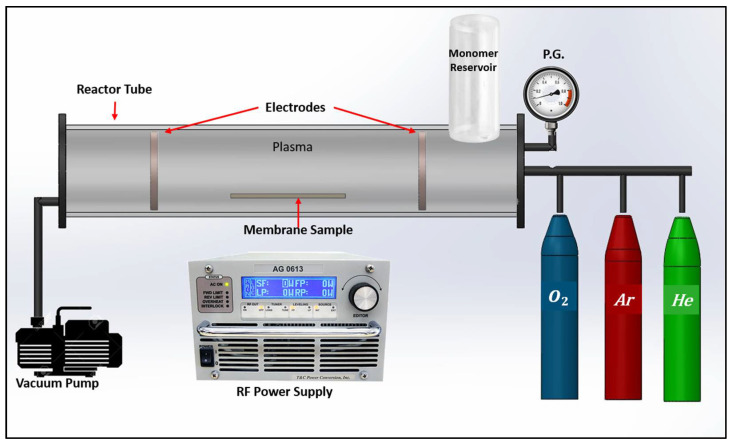
A schematic diagram of the plasma modification system.

Plasma treatment is based on the use of a lower ionization level and is generally referred to as a ‘cold’ plasma process due to the fact that it utilizes inert gases [70], where radical formation and hydrogen removal activate the polymer surface [67]. The experimental setup for plasma treatment is shown in Figure 5. Plasma interaction with the polymer membrane surface is based on four principles: cleaning, etching, cross-linking, and chemical structure modification. A lower degree of ionization solely modifies the surface of the treated substrates without changing their bulk composition, giving smooth surface modification alternatives. The plasma treatment conditions will have an impact on the physical and chemical properties of the polymer by adjusting the discharge parameters like chemical characteristics, power, and gas flowrate [71,72]. Inert gases are non-polymerizing gases, for example, argon or helium, which dissociate weak surface bonds by ion bombardment and free radical reactions. The kinetic energy of these gases can range from several electron volts; these radicals can re-react with polymer fragments when exposed to the environment [68,73]. Therefore, the membrane reacts with reactive gases like oxygen, hydrogen, carbon dioxide, nitrogen, and ammonia, resulting in a grafting reaction as chemical bonds are broken to form macromolecules like amines, amides, and oxygen-containing functional groups [70]. Combining plasma treatment with liquid macromolecule pre-adsorption enables the covalent immobilization of functional species on substrates while also preserving essential macromolecular characteristics [74,75]. PAN membranes on plasma surface treatment have been observed to reduce the skin pore size of the polymer and to preserve nitrile groups (C≡N) while introducing carboxyl (COOH) functional groups on the surface, which result in enhancing hydrophilicity [76,77]. The plasma modification method prompts oxidation, reduction, or gas mixture, and inert gases have broadened the arenas for surface modification of polymer membranes [78].

### 4.2. Graft Polymerization Technique

Graft polymerization is one of the least used methods for PAN surface modification due to its complexity [79,80]. The graft polymerization modification involves photo-induced graft polymerization, where heterogeneous polymerization-specific functionalities can be introduced onto the polymer layer [78]. Electron beam or γ-radiation is induced through plasma in modification by amides or acidic derivatives. These radiations are photo-initiated in the form of a gas phase onto the porous samples without impairing their physical and chemical properties and avoiding swelling of the sub-layer porous sample. Due to reactive monomers present on the membrane surface, a sequential step was followed using UV irradiation to avoid alteration of the graft polymer phase [81,82,83]. This technique is not preferred as it involves complex chemistry and a lengthy modification process.

### 4.3. Alkaline and Acid Hydrolysis Techniques

**Figure 6:**
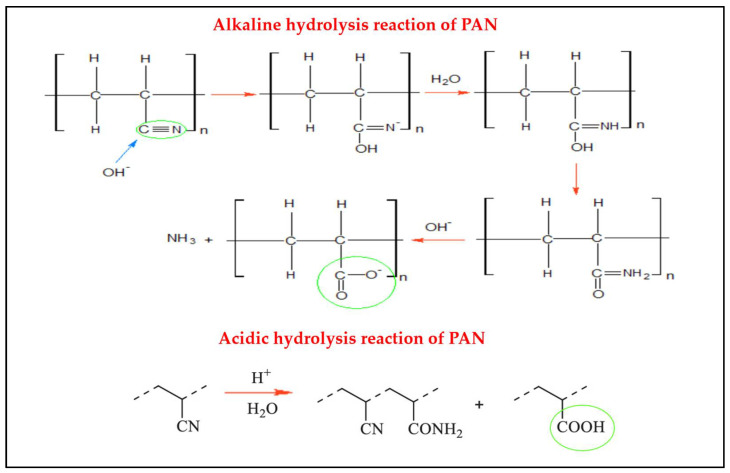
Alkaline and acidic hydrolysis reactions of PAN.

The hydrolysis technique using an alkaline solution is considered one of the simplest and cheapest methods applied to PAN membranes due to its ability in facilitating the next modification processes and increasing PAN chemical stability in common solvents [11,62,66]. By alkaline treatment, cyanide groups of PAN on the surface of the support can be converted into amide groups, which can then be converted into carboxyl groups, as shown in Figure 6. The carboxyl groups generated will increase membrane negativity charge, and hydrophilicity and will be useful for the formation of ionic and covalent bonds with amine compounds [61,84]. The establishment of an ionic bond between the two layers will act positively on water flux as well as on the chemical stability of the polyamide (PA) composite membrane [15,85]. Nevertheless, alkaline pre-treatment can lead to PAN membrane swelling and therefore reduce its roughness and pore size [84,86]. The hydrolysis process can be affected by different factors, such as alkaline species, alkaline concentration, hydrolysis time, and hydrolysis temperature [86]. As a result, selecting suitable alkaline species and hydrolysis conditions is critical for obtaining good performance from polyacrylonitrile-based membranes. On the other hand, acid hydrolysis is a topic rarely discussed in the domain of PAN membrane surface treatment. The reason behind this is that researchers claim acidic hydrolysis has a slow hydrolysis reaction rate [87]. However, when an acidic hydrolysis reaction is treated on the PAN surface, it leads to a conjugated sequence of sodium acrylates and amides co-polymer formation. This study also observed glutarimide (GI) cycles at elevated temperatures, which was a disadvantage of its instability in the reaction. In addition, acidic hydrolysis is not an ideal choice observed by researchers, as it leads to a weak linking mechanism between the polyamide and membranes [88]. It is also observed to increase the porosity of the membrane, which eventually affects the salt rejection of the membrane.

### 4.4. Click Chemistry

Another different method of altering the PAN membrane’s surface is through the use of “click chemistry”, which refers to a set of chemical reactions that are fast, selective, and easy to perform. Click chemistry is an emerging technique as an alternative to hydrolysis and grafting treatments. The click reaction has been observed to be a quantitative and orthogonal reaction that is wide in scope, generating a result in linking byproducts that does not affect the polymers. The click reaction has three different methods of modification. First, diazo reagents imidiazole-1-sulfonyl azide hydrochloride, with either a basic copper-free condition or a copper-catalyzed condition, are used as transfer reagents. Both methods result in the conversion of amino groups on the surface to azides. Secondly, carboxyl groups on the polymersomes are activated by the reaction of EDC-NHS in the presence of amino-PEG-monoazide to form amine-terminated polymersomes. Immobilization of azide-functionalized polymersomes on the membranes converts alkyne groups to triazoles [89,90]. A click chemistry reaction that is commonly used to modify the surface of PAN membranes is the copper-catalyzed azide-alkyne cycloaddition (CuAAC) reaction [90]. The reaction attacks carboxylate groups using aqueous HCl at a certain degree Celsius on the membrane, which was then readily activated using EDC/NHS coupling with propargylamine, which covalently coats functionalized PAN membrane forming a monolayer of azide-functionalized polymersomes. The thermodynamic characteristics of CuAAC have a high driving force of approximately 55 Kcal/mol, and the reaction rate of Cu increases in the order of 10^6^ orders of magnitude, leaving the reaction to be completely orthogonal. Other click chemistry reactions that have been used to modify the surface of PAN membranes include the thiol–ene reaction and the strain-promoted azide–alkyne cycloaddition (SPAAC) reaction. This reaction can be used to attach a wide range of functional groups to the surface of the membrane, allowing it to be customized for specific applications [90].

### 4.5. Static and Pore-Flowing Modifications Using Ethanolamine (ETA)

The preparation of hydrophilic membranes frequently involves surface alterations, such as physical coating and chemical modification. Chemical and physical coatings have been used to study the characteristic traits affecting the morphology and performance of the membrane. Qin et al. [68] studied the effect of static and pore-flowing conditions on PAN surface modification at different time and temperature parameters using ETA-based chemical modification to react with the nitrile groups of PAN. The static modification by ETA was observed to form a multilayer on the surface and pores, resulting in a shrinkage of pore size. The molecular weight cut-off of the membrane decreased because of the increased temperature reaction of the ETA modification, resulting in higher rejection performance. Thermal modification of static was also observed to have an effect on the membrane, which in turn benefitted the mechanical properties after modification. On the other hand, pore-flowing of ETA on the PAN membrane was found to have an impact on forming a monolayer on the membrane as it restrains the thermal motion of PAN membrane chains, causing an increased flux compared to the static procedure in which PAN chains are in a free state to respond to the thermal strain [68]. It can be concluded that the characteristics and structure of the modified PAN membrane are significantly influenced by the kinetics of the modification process [68].

### 4.6. Esterification

Esterification is another type of membrane surface modification technique [91]. This technique has two different procedures of fabrication: the first is based on pervaporation, in which an acid chemical is reacted with a catalyst directly into the liquid, and the second is based on embedding a catalyst in the catalytic layer, which is then synchronized with the pervaporation membrane. Sodium alginate mixed with MoS_2_ is used as the pervaporation separation layer on the PAN membrane, which reduces membrane swelling and has high stability. The second layer on top of the pervaporation layer is the catalytic layer, which is the main esterification reaction. Alcoholic groups are reacted with acidic groups in the presence of a catalyst, which forms the catalytic layer and completes the surface modification [91,92]. This technique is not commonly used in PAN membranes as it requires multiple stages of modifications per step, involving the use of more chemicals.

### 4.7. Hydrazine Cross-Linking

Chemical modification is the most commonly used surface modification technique, among which hydrazine hydrate is used as a cross-linking mechanism in the PAN membrane [93,94]. Hydrazine hydrate and other polyamines like ethylenediamine and diethylenetriamine are inorganic chemicals used for cross-linking. Cross-linking is a phenomenon used to enhance stability by reducing the mobility of membrane chains on membrane surfaces and improving resistance to chemical attacks. The synthesis can be carried out using a thermal treatment that cross-links PAN via the dehydrogenation and cyclization of nitrile groups [95,96]. The PAN membrane’s reaction with hydrazine results in a nucleophilic attack on nitrile groups present on the surface by bonding with a lone pair of electrons with nitrogen atoms present in the hydrazine structure. Carboxamide and carboxylate groups are the resultant cross-linking groups after hydrolysis [93,97,98]. Chemical treatment on pristine PAN membrane is observed to increase its mechanical strength, and chemical-resistant stability [98]. Table 1 shows the comparison of all the above-mentioned PAN surface modification techniques in terms of their advantages and disadvantages.

## 5. PAN-Based Porous FO Membranes

**Figure 7:**
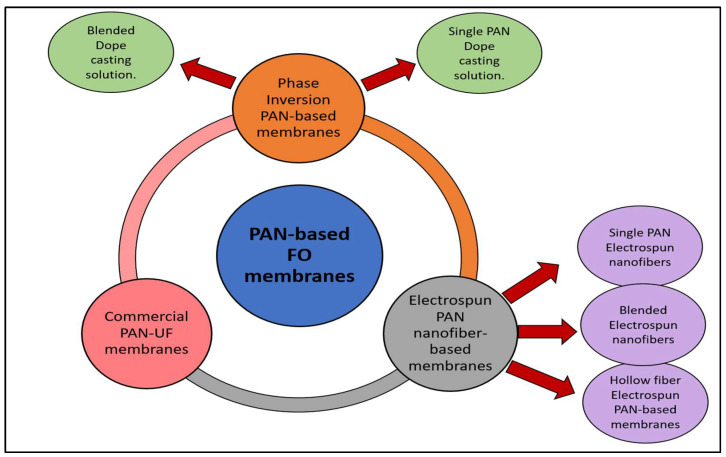
PAN-based porous FO membranes.

**Figure 8:**
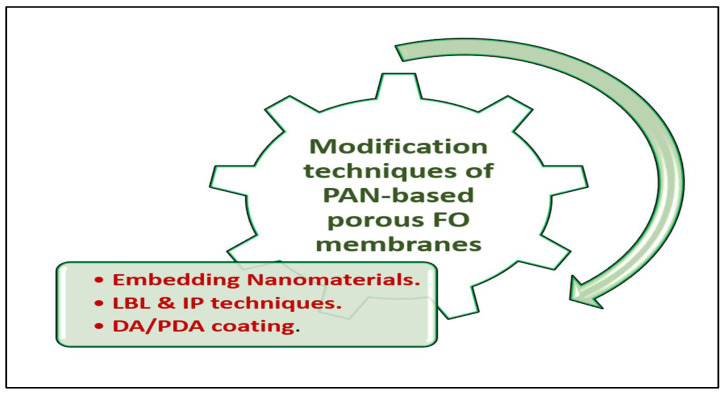
Modification techniques of PAN-based porous FO membranes.

One of the most recent advancements in PAN-based membranes is their application in the FO process, in which PAN was employed as a polymer to fabricate the substrate of simple TFC membranes and nanofiber (NTFC) membranes by the phase inversion (casting) method and electrospinning method, respectively, as shown in Figure 7. However, using commercial PAN membranes under FO conditions has rarely been studied, as will be addressed in the following. It is worth noting also that these PAN-based FO membranes modification techniques have varied between interfacial polymerization method, layer-by-layer process, embedding nanomaterials, and dopamine and polydopamine coating, as illustrated in Figure 8. Dopamine is well known for forming a polymer with high adhesive forces and excellent hydrophilic properties [101,102,103].

### 5.1. Casted PAN-Based Membranes in the FO Process

Most of the studies have focused on utilizing PAN polymer in fabricating flat sheet and hollow fiber FO membranes via the nonsolvent-induced phase separation method (NIPS) rather than fabricating FO nanofiber membranes by electrospinning, as illustrated in Table 2. This can be explained due to PAN’s relatively higher hydrophilicity compared to other commercial polymers [60]. The process of phase inversion involves casting the polymer suspension onto the support layer or backing material after mixing the polymer with the solvent. This is followed by support layer immersion precipitation [104]. The casted PAN polymeric substrates have successfully demonstrated excellent porous FO membrane performance by either using a single PAN polymer solution or blending PAN polymer with another polymer to prepare the dope cast solution.

A conventional TFC membrane based on casted PAN substrate and PA was prepared by Klaysom et al. [52], in which the selective properties have been optimized by studying the effects of different parameters during the interfacial polymerization process, such as reaction time, monomer mixture, and air-drying time. However, other approaches have studied the impact of PAN solution concentration on the morphology and FO performance of the TFC membranes [16]. A low concentration of PAN in casting solution has demonstrated higher water flux and lower RSF with low structural parameters [16,105]. While blending PAN polymer with lithium chloride (LiCl) as a pore-former agent during the NIPS method can effectively improve substrate morphology and hydrophilicity, it can also make the PA layer more dense and uniform [106]. NF-like FO membranes have been successfully prepared by forming a PA rejection layer on casted PAN substrates via interfacial polymerization [107]. These types of membranes are interestingly showing higher water permeability and higher divalent salt rejection in FO process conditions (R% > 91%) [107].

Furthermore, other studies have prepared a porous matrix substrate by incorporating nanoparticles with PAN polymer during the phase inversion method. It was proven that this technique has a high potential for increasing water flux and controlling ICP in osmotically driven membrane processes. For instance, the prepared metal–organic framework (MOF)-PAN mixed porous matrix substrate has achieved a high FO water flux of 132 LMH using deionized water (DI) and 3.0 M MgCl_2_ as an FS and DS, respectively [22]. While the prepared silica gel particles (SG)-PAN mixed matrix FO membrane has produced high FO water fluxes of more than 100 LMH by using 1 M MgCl_2_ and 0–10 mM NaCl as DS and FS, respectively [18,108]. These high-water fluxes are attributed to the ability of nanomaterials to regulate the water channels of the PAN membrane, causing finger-like hierarchical channels [109]. As a result, hydrophilicity, porosity, and pure water flux are all increased. Furthermore, using nanomaterials such as mixed CNTs has demonstrated excellent FO membrane performance when it is used as an interlayer between the commercial polyethylene terephthalate (PET) nonwoven fabric and the PAN-casted layer [110]. This sandwiched mCNT layer has caused an interconnecting porous structure to reduce the infiltration effect of PAN polymer that resists water passing [110].

Another method for incorporating NPs into PAN-casted substrates is during the IP process, where the NPs are embedded into one of the PA layer phases. This technique is considered one of the most effective ways to optimize the IP process of hydrophilic PAN support, which has a high affinity for the amine monomer required for the conventional PA selective layer IP reaction [111]. This affinity can lead to a low or prevent the crosslinking of dense and permeable PA layer formation. For this reason, several experimental works have been carried out using various materials, such as additives, co-solvents, nanoparticles, and post-treatments to tailor the selective layer structure on the top of the PAN-casted substrate. LI et al. [112] have merged carbon nanotubes (CNTs) into MPD aqueous solution. With increasing CNT concentrations, the FO performance of the fabricated TFC membrane increased in terms of water flux and RSF. The best FO performance had a water flux of 25.14 LMH and an RSF of 8.64 gMH for a 0.2 wt % CNT concentration. Moreover, Shen, Xiong, and Wang [113] have added 0–800 ppm of graphene oxide nanoparticles (GO NPs) into a 1.5 wt % MPD aqueous solution to fabricate the TFC membrane of the casted hydrolyzed PAN support layer. Incorporating GO NPs has reduced the PA layer thickness and increased support layer surface hydrophilicity, nano-channels of water molecules, and salt rejection of (81–94.6%), whereas for incorporating NPs into TMC organic solution, He et al. [114] and He, Wang, Lv et al. [115] have used sulfonated graphene oxide at metal-organic framework (SGO@UiO-66) and MOF, respectively. The fabricated TFC membranes by this method have greatly increased the membrane’s heavy-metal removal ability by more than 99.4% and salt rejection by 93.5% besides increasing their water permeability and reducing their solute leakages.

Researchers also discovered that when an aromatic hydrocarbon solvent (toluene) is employed as an organic phase in the IP process instead of aliphatic hydrocarbons, a highly selective PA layer may be formed on hydrophilic support [111]. This toluene-assisted IP (TIP) technique was used to produce highly permselective FO membranes on hydrophilic casted PAN substrates with FO performance that outperformed a commercial FO membrane (HTI-TFC): it had a double higher FO water flux and a 70% decrease in SRSF than the HTI-TFC membrane [111].

A layer-by-layer assembly technique using oppositely charged polyelectrolytes through electrostatic interaction to generate a thin selective film with a controlled structure at the nanoscale has been offered by several researchers as an alternate approach for producing high-performance casted PAN-based FO membranes [18,110,116,117,118,119,120,121,122,123,124]. These LBL-assembled membranes have experienced a high water flux. Single-skinned LBL FO membranes with a water flux of more than 100 LMH have been reported [124]. In addition, applying the LBL method to the PAN support layer has successfully formed a thinner, more hydrophilic, and denser structure of the PA selective layer, in which FO membrane selectivity performance has significantly increased [122]. The aim of depositing polyelectrolytes prior to the IP process is to improve the surface hydrophobicity of a PAN substrate that has been hydrolyzed, which allows for the creation of a more stable IP layer (i.e., minimized delamination) [125]. Under PRO mode testing, assembling three bilayers of polyelectrolytes (PAH and PSS) onto the hydrolyzed PAN support could achieve a high water flux of 55 LMH while maintaining a reasonable MgCl_2_ reverse flux of 7.5 gMH using 0.5 M MgCl_2_ as the DS. [120]. However, after UV irradiation, the membrane showed a decrease in reverse salt flux.

For further enhancement of casted PAN-based FO membranes, a few works have been reported to enhance their chlorine and antimicrobial resistance through chemical grafting and surface coating. Li et al. [126] have grafted cyclohexylamine to the PAN support layer simultaneously with IP process modification. The modified membrane showed excellent chlorine resistance with an insignificant decline in water flux. Meanwhile, Q. Liu et al. [127] and X. Liu et al. [19] have both used silver nanoparticles (Ag NPs) by an exterior surface coating method and the LBL method, respectively, to enhance PAN substrate antibacterial and antibiofilm performance. However, TFC-casted PAN-based FO membranes showed superior oil rejection (99.98%) in treating oil–water emulsions, but a higher fouling tendency compared to HTI-FO membranes owing to the higher surface roughness [128]. Blending a diamine monomer such as N-[3-(trimethoxysilyl) propyl] ethylenediamine (NPED) with MPD during the IP technique could reduce the TFC-casted PAN membrane’s surface roughness and improve their FO-fouling resistance effectively [129]. Another outperformed TFC-casted PAN membrane, with high antifouling and less ICP properties in oil–water separation, is the newly prepared double-skinned FO membrane by Duong et al. [130]. This double-skinned FO membrane consisted of a PA layer as a selective skin on top of the PAN support layer, followed by A Nexar sulfonated pentablock copolymer skin layer formed on the bottom of the support layer. Furthermore, a porous UF-like FO membrane based on hydrolyzed casted PAN substrate has performed a high rejection of poly(sodium 4-styrene-sulfonate) of about 97.5% in oil/water separation [131].

According to the great separation performance of TFC-casted PAN FO membranes, as stated above, they were utilized as a post-treatment in a study by D. Kwon et al. [132], treated the effluent produced by an anaerobic fluidized bed bioreactor (AFBR) which resulted in increased nitrogen and salt rejection. Additionally, in a study by Peng et al. [133], PAN-FO membranes have shown a high potential for separating a specific target source, such as an organic solute from salty water by creating a green tannic acid/iron selective layer instead of the conventional PA layer which leads them to have a great promise in non-desalination applications.

It is worth noting that for improving membrane performance as well as achieving a desirable membrane structure and morphology, it is crucial to control the kinetic and thermodynamic mechanisms of phase separation. A detailed effect of both mechanisms has been discussed by Ahmad et al. [134]. It was confirmed that the mass transfer rate, surface polymer concentration, and kinetics of polymer solidification—which determine the NIPS structure—are all influenced by the system temperature [134].

**Table 2:** Summary of the casted PAN-based FO membranes.

Type of PAN Membrane	MWCO of PAN Polymer	Fillers-Optimal Loading wt %	Fabrication Method	Modification Techniques	Solute Type/Applications	DS and FS	Optimum Achieved Parameters under the FO Test	References
Casted PAN substrate	(PAN, Mw 150,000 Da) was purchased from Scientific Polymer Product (Ontario, New York)		Phase inversion (16.5 wt % PAN)	Hydrolysis IP	Salt (NaCl)	FS: DI DS: 0.5 M NaCl	Casted PAN. J_w_ (PRO/FO) = 11.56/9.25 LMH J_s_ (PRO & FO) = 0.10 mole/m^2^h R% = 94.54% HPAN J_w_ (PRO/FO) = 13.88/9.25 LMH J_s_ (PRO & FO) = 0.11 mole/m^2^h R% = 89.95 %	[52]
Casted PAN substrate	(PAN, density = 1.15 g/cm^3^, molecular weight 80,000–100,000 Da) was purchased from Esfehan Polyacryl Trading Private Company (Isfahan, Iran)	-	Phase inversion (7–16 wt % PAN)	IP	Salt (NaCl)	FS: DI, NaCl (3.5 wt %) DS: 1, 2 M NaCl	PA/PAN FS: DI & DS: 1 M NaCl J_w_ = 31.3 LMH J_s_ = 5.11 gMH PA/PAN FS: NaCl (3.5 wt %) & DS:2 M NaCl J_w_ = 26.9 LMH	[16]
Casted PAN substrate	(PAN, Mw = 250,000 Da) from Hubei Chushengwei Corporation (Wuhan, China)	-	Phase inversion (4 and 16 wt % PAN)	Hydrolysis IP	Salt (NaCl)	FS: DI DS: 0.5, 2 M NaCl	4 wt % of PAN At PRO mode for DS:0.5 M NaCl J_w_ = 40.16 LMH, J_s_ = 1.22 gMH 16 wt % of PAN DS:2 M NaCl J_w_ = 44.49 LMH, J_s_ = 11.9 gMH	[105]
Casted PAN substrate	Sigma-Aldrich PAN (150,000 Da)	-	Phase inversion (12 wt % PAN)	Hydrolysis IP	Salt Simulated wastewater (Sb, Cr and aniline)	FS: DI, simulated wastewater DS: 0.5 M NaCl	TFC-PAN-1.5 wt % LiCl J_w_ = 16.5 LMH J_s_ = 2.3 gMH R% of Sb (98.2%), Cr (99.9%), and aniline (92.6%).	[106]
Casted PAN substrate	Sigma-Aldrich PAN (150,000 Da)		NIPS of PAN	IP	Salt, organic molecules,	FS: DI FS: NaCl solution (10 mmol/L), Na_2_SO_4_ solution (10 mmol/L), or SA solution (20 mg/L) DS: 1.17 mmol/L–47.00 mmol/L of neutralized Poly acrylic acid (PAANa) solution.	J_w_ = 25 LMH R% of Na_2_SO_4_ = 91.4% R% of NaCl = 21% R% of SA = 99%	[107]
MOF-PAN casted substrate	Sigma-Aldrich PAN (150,000 Da)	1 wt % of MOF particles	Phase inversion (MOF+ 18 wt % PAN)	MOF particles poured into PAN polymer matrix. PAH/PSS LBL treatment. GA crosslinking	Salt (NaCl, MgCl_2_)	FS: DI, 10, 100 mM NaCl DS: 0.1, 0.3, 0.5, 1, 3 M MgCl_2_	Control membrane in PRO mode at DS: 0.5 M MgCl_2_ and FS: DI J_w_ = 78.1 LMH Control membrane in FO mode J_w_ = 28.7 LMH MOF-based membrane in PRO mode at DS: 0.5 M MgCl_2_ and FS: DI J_w_ = 107.4 LMH MOF-based membrane in PRO mode at DS: 3 M MgCl_2_ and FS: DI J_w_ = 132.7 LMH	[22]
Casted mixed matrix PAN+ silica gel substrate	Sigma–Aldrich, PAN Mw = 150,000 Da	1.0 wt % of Silica gel particles	Phase inversion (18 wt % PAN+ Silica gel)	Hydrolysis PAH/PSS LBL. GA crosslinking	Salt	FS: DI or 10,100 mM NaCl. DS: 0.5 M MgCl_2_	FO mode J_w_ = 28.6 J_s_ = 5.8 J_s_/J_w_ = 0.20 R% by RO = 76% MgCl_2_ In PRO mode J_w_ = 77.9 J_s_ = 6.9 J_s_/J_w_ = 0.09	[18,108]
Casted PAN substrate	Sigma-Aldrich PAN (150,000 Da)		Phase inversion (Wet casting—18 wt % PAN)	LBL using polyelectrolytes. GA crosslinking	Salts (Na_2_SO_4_, MgSO_4,_ Na_3_CIT (NH_4_)_2_SO_4_ Protein (BSA and LYS)	FS: BSA and LYS DS: 1 M Na_2_SO_4_, 1 M (NH_4_)_2_SO_4_	FO mode J_w_ = 28 LMH for Na_2_SO_4_ DS. J_w_ = 40 LMH for (NH_4_)_2_SO_4_ DS	[116]
Casted HCD-decorated PAN support layer	PAN Mw of 150,000 Da, from Aladdin Industrial Corporation (Ontario, California).	10 wt % of hydrophobic carbon dots HCDs	Phase inversion- Nonsolvent-induced phase separation (NIPS) (HCD s + PAN)	IP	Salt (NaCl)	FS: DI DS: 1 M NaCl	TFC-0 in PRO mode J_w_ = 7.71 LMH J_s_ = 4.56 gMH TFC-10% HCDs in PRO mode J_w_ = 15.47 LMH J_s_ = 2.9 gMH	[109]
Casted PAN onto mCNT-PET membrane	Sigma-Aldrich PAN (150,000 Da)	TCNT and LCNT with a weight ratio of 3:1	Phase inversion of 12 wt % PAN	mCNT intermediate layer by casting onto PET Hydrolysis PEI/PAA depositing. IP of PA layer by mLBL	Salt (NaCl)	FS: DI DS: 1, 2 M NaCl	PET30-mCNT-HPAN30 At DS 1 M NaCl J_w_ = 29.02 LMH/43.5 LMH J_s_ = 9.4 gMH/11.1 gMH At DS 2 M NaCl J_w_ = 32.4 LMH/75 LMH J_s_ = 11.9 gMH/16.4 gMH	[110]
Casted PAN substrate	Sigma-Aldrich PAN (150,000 Da)	-	NIPS- Phase inversion of 12 wt % PAN	IP (Using toluene instead of hexane as a solvent for TMC)	Salt (NaCl)	FS: DI DS: 1 M NaCl	J_w_ of TFC-TIP = 34.2 LMH/44.5 LMH J_w_ of TFC-HIP = 12.9 LMH/17.0 LMH J_s_ of TFC-TIP = 5.81 gMH/8.45 gMH J_s_ of TFC-HIP = 6.96 gMH/9.35 gMH	[111]
Casted PAN substrate	Sigma-Aldrich PAN (150,000 Da)	CNTs 0.2 wt %	Phase inversion of 14 wt % PAN	Hydrolysis PEI/PAA coating IP (CNTs into MPD aqueous phase)	Salt (NaCl)	FS: DI DS: 0.5 M NaCl	J_w_ = 25.14 LMH J_s_ = 8.64 gMH J_s_/J_w_ = 0.37 g/L	[112]
Casted PAN substrate	PAN powder (Mn: 250,000 Da) was purchased from Chushengwei Chemistry Co. Ltd. (Hubei, China).	400 and 600 ppm are the optimal loadings of GO.	Phase inversion of 16 wt % PAN	Hydrolysis IP (GO into MPD aqueous phase	Salt (NaCl)	FS: DI DS: 2 M NaCl	At FO mode J_w_ = 21.6–35.4 LMH. At PRO mode J_w_ = 31.1–56.6 LMH J_s_ = 2–12 gMH R% = 81–94.6% FRR% >90 %	[113]
Casted PAN substrate	PAN powder was obtained from Chushang Co., Ltd (Hubei, China).	0.04 wt % of SGO@UiO-66	Phase inversion of 15 wt % PAN	IP (SGO@UiO-66- into TMC organic phase)	Salt (NaCl) Heavy metal removal (Cu^2+^ and Pb^2+^)	FS: DI DS: 1 M NaCl	PRO mode SGO@UiO-66-TFN Membrane (M2) J_w_ = 15 LMH J_s_ = 3 gMH J_s_/J_w_ = 0.2 g/L R% of NaCl (50 ppm) = 89.95% by RO test.	[114]
Casted Double Layer PAN	PAN powder was supplied by Chusheng Co. Ltd (Hubei, China).	0.01 wt % of MOF-801	Phase inversion of 15 wt % PAN	IP (PDA into MPD phase+ MOF into TMC phase)	Salt (NaCl) Heavy metal removal (Cd^2+^, Ni^2+^, Pb^2+^)	FS: DI DS: 1 M NaCl	FO mode R% of NaCl = 93.5%. J_w_ = 16.7 LMH J_s_ = 2.8 gMH The removal rate was 94~99.2% for Ni^2+^, Cd^2+,^ and Pb^2+^)	[115]
Casted PAN substrate	Sigma-Aldrich PAN (150,000 Da)		Phase inversion of 16 wt % PAN	Hydrolysis PEI/PAA electrostatic interaction IP by mLBL	Salt (NaCl)	FS: DI DS: 0.5 M NaCl	mLBL-10 J_w_ (FO/PRO) = 24.6 LMH/32.9 LMH J_s_ (FO/PRO) = 2.36 gMH/3.77 gMH J_s_/J_w_ (FO/PRO) = 0.10/0.11 g/L IP-TFC: J_w_ (FO/PRO) = 10.9 LMH/15.6 LMH J_s_ (FO/PRO) = 7.56 gMH/11.07 gMH J_s_/J_w_ (FO/PRO) = 0.69/0.71 g/L	[122]
Casted PAN substrate	Sigma-Aldrich PAN (150,000 Da)	-	Phase inversion of 18 wt % PAN	Hydrolysis PAH/PSS layers by LBL Assembly.	Salt (MgCl_2_, NaCl)	DS: 1 M MgCl_2_ FS: DI water or 10 mM NaCl	3# LBL FO in FO mode at FS: DI DS 1 M MgCl_2_ J_w_ = 28.7 LMH J_s_ = 0.18 mol/m^2^h J_s_/J_w_ = 6.3 mM 3# LBL FO in PRO mode at FS: DI DS 1 M MgCl_2_ J_w_ = 31.7 LMH J_s_ = 0.49 mol/m^2^h J_s_/J_w_ = 15.5 mM	[123]
Casted PAN substrate	Sigma-Aldrich PAN (150,000 Da)	-	Phase inversion of 18 wt % PAN	Hydrolysis Poly(allylamine hydrochloride) PAH/ poly(sodium 4-styrene-sulfonate PSS layers by LBL Assembly.	Salt (MgCl_2_, MgSO_4_, and Na_2_SO_4_)	FS: DI DS: MgCl_2_	FO mode J_w_ = 20–30 LMH PRO mode J_w_ = 40–60 LMH	[121]
Casted Double-skinned PAN substrate	Sigma-Aldrich PAN (150,000 Da)	-	Phase inversion of 18 wt % PAN	Hydrolysis PAH/PSS LBL assembly and crosslinking	Salt (MgCl_2_)	FS: DI DS:0.5 M MgCl_2_	xLBL3-0 J_w_ = 58.9 LMH at FS: DI, PRO mode xLBL3-0 J_w_ = 48.8 LMH at FS: 10 mM NaCl, PRO mode	[118]
Casted PAN substrate	Sigma-Aldrich PAN (150,000Da)	-	Phase inversion of 18 wt % PAN	Hydrolysis PAH/PSS LBL assembly GA crosslinking	Salt (MgCl_2_)	FS: DI DS: 3 M MgCl_2_	XLBL-3 in PRO mode J_w_ = 105.4 LMH J_s_/J_w_ = 3 mM R% by RO = 95% (500 ppm MgCl_2_)	[124]
Casted PAN substrate	PAN, Mw ~50,000 Da) was supplied by the Shanghai Jingshan Petrochemical Company (China).		Phase inversion of 18 wt % PAN	Hydrolysis LBL using PEI and PSS. Liposomes and Proteoliposomes spreading.	Salt (MgCl_2_)	FS: DI DS:2 M MgCl_2_	FO mode J_w_ = 13.2 LMH J_s_ = 3.2 gMH PRO mode J_w_ = 15.6 LMH J_s_ = 3.4 gMH	[117]
Casted PAN substrate	Sigma-Aldrich PAN (150,000 Da)	20 mg GO	Phase inversion of 12 wt % PAN	PDA/GO coating. PEI/PAA deposition IP of PA forming by LBL	Salt (NaCl)	FS: DI DS: 1 M NaCl	Nonwoven-PAN150-mLBL1 J_w_ = 10 LMH Nonwoven-PAN150-mLBL1 J_s_ = 10.4 gMH PA forming by modified mLBL method (PAN-300 thickness) J_w_ = 17.6 LMH J_s_ = 5.5 gMH	[119]
Casted PAN substrate	PAN, Mw: 150,000 Da Macklin	0.5 wt % of Cyclohexylamin	NIPS- Phase inversion of 16.7 wt % PAN	IP (Cyclohexylamine into MPD aqueous phase)	Salt sodium hypochlorite NaClO	FS: DI, 0.5 NaCl DS: 2 M MgCl_2_	For TMC-1 with 0.5 wt % of Cyclohexylamin in FO mode J_w_ = 13.2 LMH J_s_ = 9.3 gMH Salt R% = 98.5% For TFC-0 J_w_ = 12.4 LMH J_s_ = 7.1 gMH Salt R% = 98.5% The water flux of the optimal modified membrane was 10.78 LMH after chlorine exposure.	[126]
Casted PAN substrate	PAN was provided by Prof. Hui-An Tsai of Chung Yuan Christian University (Taiwan) and vacuum-dried at 80 °C in an oven before use.	-	Phase inversion of 18 wt % PAN	Hydrolysis IP	Salt Bacteria	FS: DI DS: 2 M NaCl	M-Ag J_w_ = 45 LMH in PRO mode J_w_ = 30 LMH in FO mode J_s_ = 0.32 mole/m^2^h in PRO mode J_s_ = 0.24 mole/m^2^h in FO mode	[127]
Casted PAN substrate	Sigma-Aldrich PAN (150,000 Da)	0.01 wt % of AgNPs	Phase inversion of 18 wt % PAN	PAH/PSS+ AgNPs LBL GA crosslinking	Salt Bacteria	FS: DI,10 mM NaCl DS: 0.5 M MgCl_2_	xLBL2.5-Ag (into PSS1) J_w_ = 43 LMH in PRO mode J_w_ = 18 LMH in FO mode J_s_/J_w_ = 0.07 g/L in PRO mode J_s_/J_w_ = 0.17 g/L in FO mode	[19]
Cased PAN Substrate	PAN Mw of 1,000,000 Da provided by Prof. Hui-An Tsai from Chung Yuan Christian University (Taiwan) and was vacuum-dried overnight at 60 C	-	Phase inversion of 18 wt % PAN	Hydrolysis PAH/PSS LBL assembly GA+UV crosslinking	Salt	FS: DI DS: 0.5 M MgCl_2_ DS: 0.5 M NaCl	At PRO mode For 3 LBL assembly J_w_ = 55 LMH J_s_ = 7.5 gMH	[120]
Casted PAN substrate	PAN was provided by Prof. Hui-An Tsai of Chung Yuan Christian University (Taiwan) and was vacuum-dried overnight at 60 C.	-	Phase inversion of 16 wt % PAN	Hydrolysis IP	Salt Oil	FS: oily water solutions 0 ppm, 500, 5000, 50,000, 200,000 DS: 1 M NaCl.	HPAN-TFC = 11.8 LMH for FS 200,000 ppm and DS: 1 M NaCl.	[128]
Casted PAN substrate	PAN-MWCO 250,000 Da) was purchased from Hubei Chushengwei Corporation (Hubei, China),	-	Phase inversion of 16 wt % PAN	Hydrolysis IP (MPD+NPED (N-[3-(trimethoxysilyl) propyl] ethylenediamine) + TMC crosslinking	Salt Alginate Bovine serum albumin (BSA) polysaccharides-abundant wastewater	FS: DI DS: 0.5 and 2 M NaCl	TFC-0 at FO mode DS 0.5 M NaCl J_w_ = 9.67 LMH J_s_ = 1.7 gMH R% of NaCl = 96.6% TFC-NPED 1.5 w/v% on HPAN J_w_ = 16.7 LMH J_s_ = 10 gMH R% of NaCl = 94.2%	[129]
Casted PAN substrate	PAN was provided by Prof. Hui-An Tsai of Chung Yuan Christian University (Taiwan) and was vacuum-dried overnight at 60 C.	-	Phase inversion of 14 wt % PAN. Nexar copolymer 0.05 to 2 wt %	Nexar deposition IP	Salt	FS: DI DS: 0.5 M NaCl	In PRO mode for double-skinned (TFC and Nexar copolymer) membrane J_w_ = 17.2 LMH J_s_ = 4.85 gMH In PRO mode for single-skinned (TFC) membrane J_w_ = 18.5 LMH J_s_ = 5.25 gMH In FO mode for single-skinned (TFC) membrane J_w_ = 12.8 LMH J_s_ = 3.43 gMH	[130]
Casted PAN substrate	PAN, Mw ∼150,000 Da, from Sigma Aldrich)	-	Phase inversion of 18 wt % PAN	Hydrolysis	Salt PSS	FS: DI DS: 0.1% PSS	J_w_ = 7.6 LMH R% of NaCl = 0 R% of PSS (70 kDa) = 97.5%	[131]
Casted PAN substrate	PAN Mw: 150,000 Da, from Sigma-Aldrich	-	Phase inversion of 12 wt % PAN	IP	Salt	FS: Anaerobic fluidized-bed reactor effluent DS: 0.5 or 1 M NaCl	PAN-TFC DS 0.5 M NaCl R% of NH_4_-N = 70% J_s_= 0.92 gMH J_w_ = 23.2 LMH	[132]
Casted PAN substrate	Sigma-Aldrich PAN (150,000 Da)	-	Phase inversion of 18 wt % PAN	TA/Fe coating	Salt Dye	FS: DI DS: 46.9 mM sodium polyacrylate (PAANa)	TA/Fe-PAN At FO mode R% of NaCl = 27.6% by RO test. R% of Sunset yellow = 99.5% by RO test. R% of PAANa = 96.7% by RO test. J_w_ = 22.5 LMH	[133]

### 5.2. Electrospun PAN-Based Nanofiber Membranes in the FO Process

Electrospinning is a versatile and rapidly evolving technique for the fabrication of nanofiber-based membranes. PAN has received much research attention for fabricating nanofiber membranes with highly porous layers due to its excellent compatibility, good electrospinnability, and high tensile strength after electrospinning, as shown in Table 3 [51]. Al-Furaiji et al. [17] prepared an electrospun PAN nanofiber support layer and generated a PA layer using the IP technique on top of the prepared support layer. The fabricated nanofiber TFC showed a highly stable water flux (16 LMH) and low salt rejection (4 gMH) compared to the CTA membrane (water flux = 13 LMH and salt rejection = 3 gMH). The FO process was conducted in the FO mode (active layer facing feed solution), with DI as FS and 1 M NaCl solution as DS. In another study, an aligned PAN nanofiber substrate was obtained by the electrospinning process [135]. The fabricated membrane is highly porous with high hydrophilicity and a slit-shaped pore structure, which mitigates the ICP effect during the FO process. It was also noted that the PA layer created on top of the aligned PAN nanofiber was thinner than the one created on top of the randomly oriented nanofiber substrate. As a result, the resultant TFC-aligned PAN nanofiber exhibited impressive performance in terms of water flux (50.7 LMH and 62.9 LMH) and low SRSF in FO and PRO orientation modes using 1 M NaCl solution and DI water as a DS and FS, respectively [135]. Moreover, an electrospun nanofibrous composite PAN membrane with an NF-selective layer has been successfully performed under the FO process using branched PEI as a DS and DI water as a FS [136]. The prepared membrane could result in a significant increase in water flux (14 LMH) with very little or no reverse solute diffusion. It was also found that the performance of TFC-PAN nanofiber can be highly affected by the types of PA layer monomers used during the IP crosslinking reaction [137]. Forming a PA layer on top of PAN nanofiber using MPD and TMC showed a lower salt flux of 2.53 gMH compared to PEI and an isophthaloyl chloride (IPC)-based PA layer (13.55 gMH). However, the water flux has been compensated for the PEI and IPC-based PA layer, achieving 31.51 LMH compared to the MPD and TMC- based PA layer (28.15 LMH) due to the thin-selective layer formed by PEI and ICP [137].

The promised osmotic performance with a finely tuned pore structure of the support layer was achieved using hydrophilic PAN polymer for fabricating TFC hollow fiber membranes, via dry jet–wet spinning [138]. The prepared TFC hollow fiber membrane showed high water fluxes of 36.6 LMH and 24.71 LMH in the PRO and FO modes, respectively [138]. In another work, a patent was achieved using a tubular nanofiber made of electrospun PAN as a support layer for TFC FO membranes. This membrane had a high water flux of 395.1 LMH and a low RSF of 0.38 gMH under the FO test using 0.5 M as a DS and DI as an FS [139]. Through this, PAN has demonstrated great potential in being a suitable substrate polymer for TFC flat sheets and hollow fiber membranes for FO applications. Moreover, another study by Chi et al. [140] used a hydrophobic electrospun PAN nanofiber to provide excellent mechanical strength for designing a three-layer composite FO membrane. The three layers were composed of hydrophobic PAN nanofiber, followed by hydrophilic CTA nanofiber, and then PA layer formation. However, the PAN nanofiber was coated with dopamine (DPA)/polyethyleneimine (PEI) to create a positively charged surface and increase the hydrophilicity [141]. Under FO conditions, with DI as FS and 0.1 M chitooligosaccharide (COS) as DS, the results revealed low RSF and fouling effects but a reasonably high water flux (8 LMH and 4 LMH) in PRO and FO modes, respectively [140]. Furthermore, a TFC-electrospun PAN nanofiber membrane had shown a superior rejection ratio as high as 99.8% in treating tetracycline (TC) wastewater treatment through an FO−membrane distillation (MD) hybrid process, and that was confirmed by Pan et al. [142]. This fabricated nanofiber also achieved a high-water flux (57 LMH) compared to commercial FO membranes of HTI-CTA (9 LMH) and HTI-TFC (13 LMH), due to its high perm-selectivity and low structural parameters.

It has been noticed that preparing PAN nanofiber by electrospinning is not suitable for the IP process directly. As a consequence, some researchers have improved the binding strength of PAN nanofibers with a PA selective layer, using a thin chitosan (CS) sublayer that was cast on top of hydrolyzed electrospun PAN nanofiber before carrying out the PA formation [23,143,144]. Besides improving the interfacial strength between the PAN nanofiber mat and active layer, this technique has also successfully improved the mechanical strength of the PAN support, achieving a tensile strength of 28.12 MPa and a Young’s modulus of 90.66 [143]. In addition, with the optimal CS concentration (3.5%), the PAN/CS TFC nanofiber membrane has achieved 97 % rejection of NaCl salt, 55.05 LMH water flux, and 0.93 gMH reverse salt flux when it is operated in FO mode using DI as FS and 1.5 M NaCl as DS [143]. In another study, a PAN/CS composite nanofiber membrane achieved a water flux of 85.4 LMH, 0.73 gMH RSF, and 97.4% salt rejection when the CS concentration was 3.8% [23].

For more FO membranes enhancement, several studies have fabricated a new class of TFC-FO membranes via blending hydrophilic PAN polymer with another polymer material to merge their beneficial characteristics and create new electrospun nanofiber substrates. For example, through the electrospinning technique, polyvinylidene fluoride/polyacrylonitrile (PVDF/PAN) electrospun nanofiber was fabricated by Kallem et al. [54] and used as a substrate for the TFC-FO membrane. PVDF was used as a mechanical support and PAN as a hydrophilic modifier and ICP mitigator. After thermal treatment, the newly designed membrane demonstrated high-performance concerning water flux (33.3 LMH in FO mode and 42.3 LMH in PRO mode) and achieved a low SRSF value of 0.27 g/L. In another experimental work, a similar concept was followed by Shokrollahzadeh and Tajik [145] and Kallem et al. [53] to synthesize a polysulfone (PSf)/PAN and polyethersulfone (PES)/PAN blend of nanofibrous substrates, respectively. Which has been then used for creating a TFC-FO membrane with an ultrathin PA layer. Excellent selective and permeable FO performance was achieved for the blended electrospun membranes. PSf/PAN TFC blended nanofiber had a higher water flux of 38.3 LMH and a lower RSF of 10.1 gMH in PRO mode compared to the PSf/PAN TFC membrane prepared using the conventional phase inversion technique (J_w_ = 12.6 LMH and J_s_ = 11.6 gMH) [145]. While PES/PAN blended nanofiber achieved a greater water flux of 42.1 LMH and a lower RSF of 11.4 gMH in FO mode compared to the traditional TFC membrane (J_w_ = 13.8 LMH and J_s_ = 8.83 gMH) [53]. Unlike the studies stated above, which have blended PAN with hydrophobic polymers for fabricating nanofibers. Bui and McCutcheon [146] have designed a blended electrospun nanofiber from two hydrophilic polymers. PAN and cellulose acetate (CA) have perfectly formed hydrophilic nanofiber-supported TFC membranes with a highly perm-selective attitude, water flux of 44 LMH, and RSF of 4 gMH [146]. Furthermore, recent advances in enhancing electrospun PAN membrane performance are incorporating specific nanoparticles. Bui and McCutcheon [21] have incorporated silica nanoparticles (SiNPs) during the PAN electrospinning process. The resulting thin-film nanocomposite (TFN) nanofiber membrane obtained high water flux values of 58 LMH and 82 LMH in FO and PRO mode orientations, respectively, due to the porosity and increased water uptake capacity of the NPs. However, TFN’s reverse salt flux values were 8.7 gMH and 11.5 gMH in both modes. Nanoparticles can also be used for mitigating FO membrane anti-biofouling. Silver nanoparticles (AgNPs) as an antimicrobial agent have been utilized by Pan et al. [51] during the fabrication of PAN electrospun nanofiber mat. In another way, nanoparticles have been confirmed as a modifier for adjusting PAN electrospun support characteristics when it is used as an interlayer between the electrospun PAN support layer and PA-selective layer. Thus, several materials, such as carbon nanotubes (CNTs) [20], natural mineral–halloysite nanotubes (HNTs) [147], polydopamine nanoparticles (PDA NPs) [148], and graphene oxide nanoparticles (GO NPs) [149], have been applied as an intermediate modification layer and have perfectly affected the membrane morphology by modifying the pore size and the surface roughness. Moreover, this technique has greatly enhanced the selection properties of the fabricated nanofiber TFN membrane under the FO treatment process by achieving elevated water flux and low reverse salt flux, as illustrated in Table 3. For the effect of NPs on the PA-selective layer, N. Li et al. [23] have embedded multi-walled carbon nanotubes (MWCNTs) into the PA layer, which reduces their crosslinking degree on top of the electrospun PAN support layer. The addition of MWCNTs showed higher water flux values without sacrificing RSF. In addition, embedding nanoparticles into a selective layer, such as polyhedral oligomeric silsesquioxane POSS, has improved PAN membrane antifouling properties in the FO process [150]. The same POSS nanoparticles were used in another study to prepare the outer selective layer for PAN hollow fiber membranes by spinning, resulting in an integrally macrovoid-free and delamination-free dual-layer membrane [151]. This newly prepared hollow fiber membrane exhibited 31.37 LMH as water flux in the FO process. While no reversible solute (sucrose) flux was observed for the greener hollow fiber membranes based on polyacrylonitrile (PAN) and less toxic mixtures of ionic liquids [152].

**Table 3:** Summary of PAN-based nanofiber FO membranes.

Type of PAN Membrane	MWCO of PAN Polymer	Fillers-Optimal Loading wt %	Fabrication Method (PAN or Blended Nanofiber).	Modification Techniques	Solute Type/Applications	DS and FS	Achieved Parameters under FO Test.	Voltage	References
Nanofiber PAN	Mw = 150,000 Da supplied by Macklin, Shanghai, China.	-	Electrospinning (PAN nanofiber)	IP	Salt (NaCl)	FS: DI DS: 1 M NaCl	At FO mode. J_w_ = 16 LMH J_s_ = 4 gMH	30 kV	[17]
Nanofiber PAN	PAN Mw = 70, 000 Da) supplied by Chushengwei Chemistry Co. 132 Ltd. (Hubei, China).	-	Electrospinning (14 wt % PAN nanofiber)	IP	Salt (NaCl)	FS: DI DS: 1 M NaCl	At FO mode for PAN-1500 rpm J_w_ = 50.7 LMH J_s_/J_w_ = 0.13 g/L At PRO mode for PAN-1500 rpm J_w_ = 62.9 LMH R% = 90.3% by RO test.	20 kV	[135]
Nanofiber PAN	Sigma-Aldrich PAN (150,000 Da)	-	Electrospinning (9 wt % PAN nanofiber)	IP (PEI+TMC)	Salt, TOC	FS: DI, TOC DS: 10 wt % PEI	J_w_ (PRO/FO) =24/14 LMH J_s_ = 0.7~1.0 gMH R% of NaCl 30–60%	17–19 kV	[136]
PAN nanofiber	Sigma-Aldrich PAN (150,000 Da)	-	Electrospinning (10 and 12 wt % PAN nanofiber)	IP	Salt (NaCl)	FS: DI DS: 1 M NaCl	FO mode p-TFC membrane J_w_ = 31.51 LMH J_s_ = 13.55 gMH m-TFC membrane J_w_ = 28.15 LMH J_s_ = 2.53 gMH	30 kV	[137]
PAN Hollow fibre membrane	Sigma-Aldrich PAN (150,000 Da)	-	Dry-jet-wet spinning (16 wt % PAN nanofiber)	IP	Salt (NaCl)	FS: DI DS: 1 M NaCl	J_w_ (PRO/FO) = 36.6/24.71 LMH J_s_ (PRO/FO) = 18.75/19.20 gMH J_s_/J_w_ (PRO/FO) = 0.57/0.79 g/L	Syringe pump flow rate of 4 mL/min.	[138]
PAN Tubular nanofiber	Not available		Electrospinning (10% PAN nanofiber)	Hydrolysis IP	Salt (NaCl)	FS: DI DS: 0.5 M	J_w_ = 395.1 J_s_ = 0.38 J_s_/J_w_ = 0.001 g/L	20 kV	[139]
Nanofiber PAN+CTA	PAN, 500,000 Da supplied by Shanghai Jinshan Petroleum Co. Ltd. (China).	-	Electrospinning (Blended nanofiber of PAN + CTA)	IP Dopamine hydrochloride DPA+ PEI coating	Salt (NaCl) chitooligosaccharide (COS)	FS: DI DS: 0.1 M chitooligosaccharide (COS), 1 M NaCl	DS: as NaCl J_w_ (PRO/FO) = 34.2/25.1 LMH J_s_ (PRO/FO) = 9.6/6.1 gMH DS as COS J_w_ (PRO/FO) = 8.2/4.1 LMH J_s_ = 0 gMH	14–15 kV	[140]
Nanofiber PAN	PAN Mw = 90,000 Da supplied by Kunshan Hongyi Plastic Co. (Suzhou, China).	-	Electrospinning (10 wt % PAN nanofiber)	IP	Salt (NaCl). Antibiotic wastewater (tetracycline hydrochloride TC wastewater).	FS: DI DS: 1 and 2 M NaCl	PA/PAN-eTFC at FO J_w_ = 41 LMH J_s_ = 8.7 gMH At PRO J_w_: 57 LMH at 2 M DS J_s_: 20 gMH at 2 M DS	15 kV	[142]
Nanofiber PAN	PAN Mw = 150,000 Da supplied by Shaoxing Gimel Advanced Materials Technology Co., Ltd (China).	CS-3.5%	Electrospinning (10 and 12 wt % PAN nanofiber)	Hydrolysis CS sublayer casting. IP	Salt (NaCl).	FS: DI DS: 1.5 M NaCl	For CS-3.5 J_w_ in PRO/FO: 64.88/55.05 LMH J_s_ in PRO/FO: 2.12/0.93 gMH R% of salt = 97%	30 kV	[143]
Nanofiber PAN	PAN Mw = 150,000 Da supplied by Zhongna Technology Co. Ltd (China).	CS- 3.8% 0.05 wt % of OMWCNTs	Electrospinning (12 wt % PAN nanofiber)	Hydrolysis CS casting sublayer. IP (OMWCNTs into MPD aqueous phase)	Salt (NaCl) Bovine serum albumin (BSA)	FS: DI DS: 0.5 M NaCl	PA-3.8-OMWCNTs at FO mode J_w_ = 96.9 LMH J_s_ = 0.73 gMH R% of NaCl = 97.4% when FS = 15 mM NaCl	18 kV	[23]
Nanofiber PAN	PAN, 500,000 Da) supplied from Shanghai Jinshang Petroleum Co. Ltd. (China).	CS solution for TFC-CS-PAN-3 contains 1.75 g of CS For TFC-CS-PAN-4 contains 2 g of CS	Electrospinning (10 wt % PAN nanofiber)	CS+ GA crosslinking IP	Salt (NaCl)	DS: 2 M glucose FS; 0.1 M NaCl	TFC-CS-PAN-3 J_w_ = 11.9 LMH R% of NaCl = 66% TFC-CS-PAN-4 J_w_ = 10.7 LMH J_s_ = 8.9 gMH salt flux R% of NaCl = 83.5% by RO test.	15–16 kV	[144]
Blended Nanofiber PVDF+PAN	Sigma-Aldrich PAN (150,000 Da)	-	Electrospinning (Blended 18–20 wt % PVDF+ 0–10 wt % PAN nanofiber)	IP	Salt (NaCl)	FS: DI DS: 1 M NaCl	Optimal FO condition J_s_/J_w_: 0.27 g/L J_w_: 33.3 LMH J_s_: 7.8 gMH	19–21 kV	[54]
Blended Nanofiber PSf/PAN	Sigma-Aldrich PAN (150,000 Da)	-	Electrospinning (Blended 20 wt % Psf + 15 wt % PAN nanofiber)	IP	Salt (NaCl, KCl, MgCl_2_, and MgSO_4_)	FS: DI DS: 1 M NaCl, 1.06 M KCl, 0.59M MgCl_2_, and 1.85M MgSO_4_	PAN/PSf NTFC at PRO mode J_w_ = 38.3 LMH J_s_ = 10.1 gMH PAN/PSf TFC at PRO mode J_w_ = 12.6 LMH J_s_ = 11.6 gMH	20 kV	[145]
Blended nanofiber PES/PAN	Sigma-Aldrich PAN (150,000 Da)	-	Electrospinning (Blended 18, 20, 22 wt % PES + 0–10 wt % PAN nanofiber)	IP	Salt (NaCl)	FS: DI DS: 1 M NaCl	NTFC-10 at FO mode J_w_ = 42.1 LMH J_s_/J_w_ = 0.27 J_s_ = 11.4 gMH NTFC-10 at PRO mode J_w_ = 52.2 LMH J_s_/J_w_ = 0.24	21 kV	[53]
Blended nanofiber CA/PAN	Sigma-Aldrich PAN (150,000 Da)	-	Electrospinning (Blended CA +PAN nanofiber) Ration of PAN/CA = 0/10 to 2/8, 5/5, 8/2, and 10/0	IP	Salt (NaCl)	FS: DI DS: 1.5 M NaCl	FO mode for PAN-20CA J_w_ = 44 LMH J_s_ = 4 gMH PRO mode J_w_ = 55 LMH J_s_ = 11.5 gMH	28.5 kV	[146]
SiO_2_/PAN nanofibrous	Sigma-Aldrich PAN (150,000 Da)	15 wt/wt % of SiO_2_ NPs	Electrospinning (SiO_2_ NPs +12 wt % PAN nanofiber)	IP	Salt (NaCl)	FS: DI DS: 1 M NaCl	At FO mode. J_w_: 58 LMH J_s_: 8.7 gMH J_s_/J_w_: 0.15 g/L At PRO mode. J_w_: 82 LMH J_s_: 11.5 gMH	28.5 kV	[21]
AgNO_3_ /PAN nanofibrous	PAN, Mw = 90,000Da) was purchased from Kunshan Hongyu Plastic Co., Ltd. (China).	2 wt % of AgNO_3_	Electrospinning (AgNO_3_+ 10 wt % PAN nanofiber)	IP	Salt (NaCl)	FS: DI DS: 0.5 M NaCl	J_w_:(PRO/FO) = 29.21/21.58 LMH J_s_:(PRO/FO) = 17.5/7.5 gMH	15 kV	[51]
PAN nanofiber	PAN, Mw = 250,000 Da was purchased from DuPont Co., Ltd.	0.2 wt % Dopamine modified HNTs	Electrospinning (14 wt % PAN nanofiber)	Dopamine coating Vacuum filtrating modified HNTs. IP	Salt (NaCl)	FS: DI DS: 0.5 M–2 M NaCl	At FO mode and DS 1 M J_w_ = 28 LMH J_s_ = 2.8 gMH At PRO mode and DS 1 M J_w_ = 45 LMH J_s_ = 4.2 gMH	17 kV	[147]
PAN nanofiber	PAN, Mw = 150,000 Da) was purchased from Kunshan Hongyi Plastic Co., Ltd. (China).	2 wt % of CNTs	Electrospinning (12 wt % PAN nanofiber)	CNTs interlayer IP	Salt (NaCl)	FS: DI DS: 1 M NaCl	PAN-CNTs-2 In PRO- J_w_ = 61.6 LMH In PRO- J_s_ = 7.7 gMH In FO- J_w_ = 49.2 LMH In FO- J_s_ = 7.2 gMH	15 kV	[20]
PAN nanofiber	PAN, Mw = 250,000 Da) were obtained from China National Petroleum Corporation	6 mL of PDA NPs	Electrospinning (17 wt % PAN nanofiber)	PDA NPs vacuum filtered as an interlayer. IP	Salt (NaCl) Heavy metal removal (Cu^+2^)	FS: DI DS: 1 M NaCl	TFC-6 mL PDA NPs J_w_ = 28.5 LMH R% of Cu^+2^ = 97%	16 kV	[148]
PAN nanofiber	PAN powder from Sigma-Aldrich	28 μg/cm^2^ of GO	Electrospinning (10 wt % PAN nanofiber)	GO vacuum filtered as an interlayer. IP	Salt (NaCl)	FS: DI DS: 1 M NaCl	At FO mode SRSF: 0.26 g/L J_w_: 32.7 LMH J_s_: 8.5 gMH	21 kV	[149]
As spun-PBI–POSS/PAN nanofiltration hollow fibre membranes,	PAN copolymer was provided by Prof. Hui-An Tsai from Chung Yuan Christian University, Taiwan	0.5 wt % of POSS	Spinning (As-spun PBI–POSS/PAN)	(PAN for inner substrate layer) (PBI and POSS for outer selective layer)	Salt (MgCl_2_, NaCl)	FS: DI DS: 2 M MgCl_2_	FO mode for As-spun PBI–POSS/PAN J_w_ = 17.7 LMH J_s_ = 27.6 gMH J_s /_/J_w_ = 1.6 g/L FO mode for Annealed PBI–POSS/PAN J_w_ = 12.6 LMH J_s_ = 8.8 gMH J_s /_/J_w_ = 0.7 g/L	Outer dope flow rate = 6 m/min	[150]
As spun-PBI–POSS/PAN dual-layer hollow fibre membranes.	PAN copolymer was provided by Prof. Hui-An Tsai from Chung Yuan Christian University, Taiwan	0.5 wt % of POSS	Spinning (As-spun PBI–POSS/ 16 wt % PAN)	(PAN for inner substrate layer) (PBI and POSS for outer selective layer)	Salt (MgCl_2_ and NaCl)	FS: DI DS: 2 M MgCl_2_ for FO process DS: 1 M NaCl for PRO process	FO process J_w_ = 31.37 LMH R% of MgCl_2_ = 92.3 % R% of NaCl = 81.6 %	Outer dope flow rate = 6 m/min.	[151]
Hollow fibre PAN/ Ionic liquid	PAN, Mw = 324,000 Da.	-	Spinning (12 wt % PAN) + 80 wt % Ionic liquid	IP	Sucrose	FS: DI DS 1 M and 2 M sucrose	J_w_ = 6.7 LMH J_s_ = 0 gMH	Flow rate of dope solution = 2.8 mL/min Flow rate of inner coagulant = 3 mL/min	[152]

### 5.3. Commercial PAN-Based Membranes in FO Test

In the literature, very little work has been carried out on developing commercial polyacrylonitrile membranes to prepare a thin-film composite forward osmosis membrane, as shown in Table 4. L. Yang et al. [153] have fabricated several polyamide layers on top of the commercial PAN-UF substrate surface with a pore size of 0.1μm via the conventional interfacial polymerization method and LBL method, after it was hydrolyzed by NaOH. The optimal FO performance was achieved at eight assembly cycles, where the highest water flux of 14.4/7.8 LMH (PRO/FO) and the lowest reverse salt flux of 10.0/5.4 gMH (PRO/FO) were obtained. In another study, the TFC membrane of a commercial PAN substrate exhibited a favorable water permeability flux of 16.1, LMH, and an extremely low reverse salt flux (1.25 gMH). Within this, polyethyleneimine (PEI) was utilized as an interlayer prior to the IP reaction to tailor the PA layer, which thus improved TFC-PAN membrane perm-selectivity performance due to the formed denser, thinner, and smoother PA layer [154]. The same modification technique was followed by Farman et al. [155], in which the modified membrane showed a reasonable performance for concentrating the orange juice via the FO process. However, introducing graphene quantum dots (GQDs) as a derivative of graphene oxide into the PEI aqueous phase during the IP crosslinking technique resulted in a TFC-PAN membrane with exceptional antifouling capabilities and high rejection performance. Therefore, under the FO test, the modified commercial PAN substrate exhibited a water flux of 12.9 LMH and a comparable RSF of 1.41 gMH, when DI and MgCl_2_ were used as the FS and DS, respectively [156]. Therefore, it can be said that after using the proper modification techniques, the PAN substrate layer has the ability to function as a good support layer to create TFC membranes with excellent performance under FO process conditions.

## 6. Performance Comparison of PAN-Based FO Membranes

Most of the PAN-porous-based FO membranes were fabricated at lab scale mainly through the phase inversion method, as indicated by 33 studies. On the other hand, few membranes were prepared by electrostatic spinning to prepare the PAN nanofiber support, and this is represented by 24 studies; however, the number of studies that have used commercial PAN-based UF membranes under the FO process was limited to four studies only. By comparing the FO performance of the three types of PAN-based membranes, electrospun PAN-based nanofibers membranes exhibit great potential to compete with conventional phase inversion PAN-based membranes. As shown in Table 2 and Table 3, most of the nanofiber PAN-based membranes show higher water permeable fluxes in the range of (11–97 LMH) under the FO orientation mode than the phase inversion PAN-based membranes (7–40 LMH) and even greater than those of commercial membranes. This is mainly attributed to the relatively high porosity, controllable pore size and pore size distribution, and fully interconnected open pore structures, which permit a shorter path for the diffusion of molecules [149]. Also owing to their low structural parameters (S), on the other hand, commercial phase inversion PAN support layers showed lower water flux by the FO process due to their thickness, which inhibits diffusion [157]. Similarly, the TFC membranes, based on the PAN supports fabricated via the conventional phase inversion approach, result in significant ICP because of the high S parameter value compared to the nanofiber PAN-based membranes, which reduced effective osmotic driving force and created severe flow resistance within thick and dense support layers. For example, Shokrollahzadeh and Tajik [145] have claimed that the fabricated PAN/PSf NTFC membrane exhibited a higher water flux of (38.3 LMH) and a better performance of the membrane than the PAN/PSf TFC membrane (14.3 LMH) due to the considerable decreasing of the S parameter (0.34 mm) and the ICP effect.

**Figure 9:**
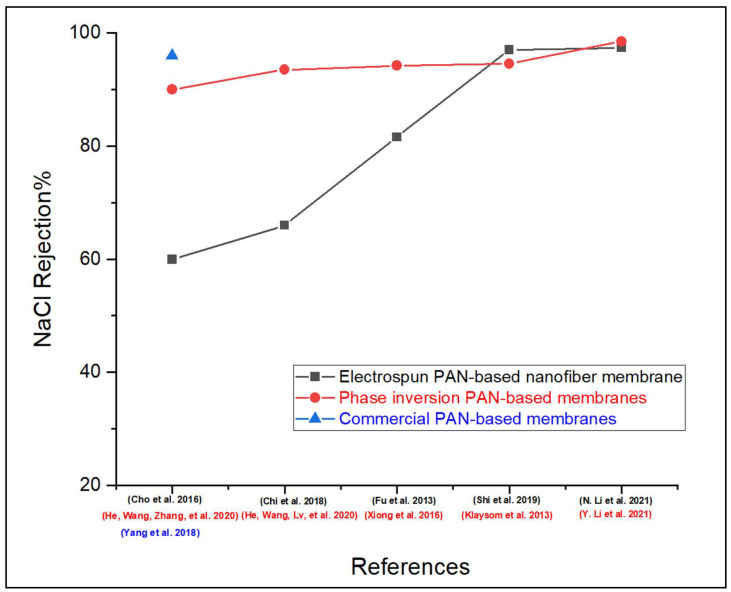
Range of NaCl salt rejection performance of different PAN-based FO membranes [23,52,114,115,126,129,136,143,144,151,153].

In addition, PAN nanofibers’ extremely large surface area enables them to maximize surface functionalization and hybridization by chemical substances and nanostructures, respectively. As a result of their surface functionalization, the applications for these nanofibers will significantly increase. However, with respect to the salt rejection performance, the TFC-phase inversion PAN-based membranes were showing better rejection performance (>90%), as shown in Figure 9, because of the highly crosslinked PA layer, unlike electrospun nanofiber PAN substrates which suffer from an unstable PA layer due to the poor adhesion between the PA and nanofibers [48,158,159,160].

It is worth mentioning that the different water flux values of the same type of PAN-based membrane are related to the modification technique procedure using various additives and nanomaterials as well as to the applied operation parameters and conditions, such as hydrodynamic conditions and flow direction of the FS and DS as driving forces of water transportation during the process operation. Table 5 exhibits the comparison of the most commonly utilized FO-based membrane fabrication and modification techniques. The active layer of a high-performance TFC FO membrane should have outstanding permeance and great selectivity. Meanwhile, to reduce ICP, its support layer must be thin, hydrophilic, and extremely porous. 

## 7. Conclusions and Perspectives

Polyacrylonitrile polymer, with its unique properties, has confirmed its ability to fabricate FO support membranes as well as its applicability to a variety of module types and consequently FO applications. This is because PAN is more easily modifiable than other polymeric materials used for membranes. In addition, PAN contains nitrile groups that enable a variety of chemical processes to enhance membrane structure and morphology, including hydrolysis, cyclization, and amination. Other than the polymer properties, the preparation conditions could also affect membrane structure and morphology. This paper has shown that different types of FO membranes have been fabricated based on the PAN polymer via phase inversion and electrospinning techniques. Numerous laboratory experiments have also been conducted to modify the physicochemical properties and performance of these PAN-based membranes, including membrane structure, permeability, mechanical strength, hydrophilicity, porosity, and surface charge, using various additives such as nanomaterials and polyelectrolytes during their fabrication stage or IP process. All lab-made PAN-based FO membranes have exhibited great potential for application under FO operating conditions at the lab scale. Their performances were described as achieving good water flux, comparable low reverse solute flux, high salt rejection, and high antifouling and mechanical strength. However, it should be noted that most of the nanofiber PAN-based membranes have demonstrated larger water permeable fluxes under the FO orientation mode compared to the phase inversion PAN-based membranes and even greater than those of commercial membranes in the range of (11–97 LMH). Although electrospun-PAN nanofiber-based substrates can provide significant potential in the FO process owing to their lower structural parameters and porous structure compared to PI PAN-based substrates, more research is required to improve their mechanical characteristics, optimize the pore size of ENs PAN substrates, and prevent the PA layer from delaminating from the ENs PAN substrates. Additionally, despite the significant increase in research on PAN-based FO membranes, their anti-fouling performance is still limited and needs to be addressed, as well as their performance at full-scale and pilot-scale systems, which still needs to be tested to better understand their FO performance.

## Figures and Tables

**Table 1:** Surface modification techniques of PAN membrane.

PAN Surface Modification Techniques	Pros	Cons	References
Plasma treatment	Stable water flux. Anti-fouling. Smooth surface roughness.	Laboratory cost-effective. Complex modification.	[71,99]
Graft polymerization	Anti-swelling. Unaltered chemical and physical properties. Chemical resistance.	Complexity Time-consuming.	[83]
Alkaline hydrolysis	Stable water flux. Fast cross-linking reaction. Sustain high temperatures. Increase salt rejection. Cost effective.	Physical change in the backing layer. Reduce pore size.	[86]
Acidic hydrolysis	Slow reaction. Cost effective.	Weak bond formation.	[88]
Click Chemistry	Rapid synthesis and high yield. High chemical resistance.	Complex chemistry. Alkyne homocoupling.	[90,100]
Static And Pore flowing modification using Ethanolamine.	Multilayer surface formation. Uniform layer. Increase surface hydrophilicity. Enhanced mechanical properties.	Lower selectivity.	[68]
Esterification.	Increase membrane stability. Anti-swelling properties.	Requires multiple stages. More chemical usage.	[91,92]
Hydrazine Cross-linking.	Increase membrane stability. Improve resistance to chemical attacks. Increase membrane mechanical strength.	Complex reaction.	[93,97]

**Table 4:** Summary of commercial PAN-based membranes under FO test.

Type of PAN Membrane	MWCO KDa	Fillers-Optimal Loading wt %	Fabrication Method	Modification Techniques	Solute Type/Applications	DS and FS	Achieved Parameters under FO Test	References
Commercial PAN membrane	Mean pore size of 0.1 μm	-	Commercial membrane Supplied by Beijing Ande Membrane Technology, China.	Hydrolysis LBL IP	Salt (NaCl)	FS: DI DS: 1 M NaCl	Optimal conditions for LBL-8. J_w_ PRO/FO: 14.4/7.8 LMH J_s_ PRO/FO = 10/5.4 gMH R% = 96% by RO test.	[153]
Commercial PAN membrane	NA	-	NA	Hydrolysis electrostatic interaction using PEI. IP.	Salt (MgCl_2_)	FS: DI DS: 2 M MgCl_2_	Optimal conditions for TFC-PEI-1.5 membrane at FO mode. J_w_ = 16.1 LMH J_s_ = 1.25 gMH	[154]
Commercial PAN UF- membrane	(PAN-50,000 Da)	0.05 wt % GQDs	Commercial membrane Supplied by Suntar Membrane Technology (Xiamen, China).	Hydrolysis IP (GQDs into PEI aqueous phase).	Salt (MgCl_2_) Humic acid BSA	FS: DI DS: 0.5 M MgCl_2_	At FO mode. J_w_ = 12.9 LMH J_s_ = 1.41 gMH	[156]
Commercial PAN UF- membrane	NA	-	NA	Hydrolysis PEI interlayer coating. IP	CaCl_2_ Glucose Sodium acetate (CH_3_COONa)	FS: DI DS: 5% CH_3_COONa with CaCl_2_	At PRO mode J_w_ = 23.9 LMH J_s_ = 6.64 gMH	[155]

**Table 5:** Comparison of the most employed FO membrane fabrication and modification techniques.

	Technique.	Pros	Cons	References
Fabrication technique	Phase inversion	Uniform thickness distribution. Good flatness. High flux.	Limited to specific polymers. Depend on many parameters. No- uniformity in pore size distribution. Uncontrolled pore size and pore diameter. Low mechanical strength. Time-consuming technique. High surface roughness.	[161,162,163,164]
	Electrospinning	Large surface area-to-volume ratio. High porosity. Formation of interconnected pores. Easily combined with different materials. High mechanical strength. High flux.	Depend on many parameters. Jet instability. High-voltage power supply. High surface roughness. Require post-treatment.	[163,164,165]
Modification technique	Layer By Layer assembly	Finely tuneable. Control membrane thickness, roughness, and surface charge.	Time-consuming. Require an appropriate crosslinker. Not appropriate for large-scale production.	[163,166,167]
	Interfacial polymerization	Simple technique. High anti-fouling properties. High retention. Low surface roughness. Easily combined with different materials. High surface charge. Low width of pore size distribution.	Low Flux. At the industrial manufacturing scale, it is not economically viable and environmentally friendly because of the high chemical demand.	[168,169]
